# FSH: fast spaced seed hashing exploiting adjacent hashes

**DOI:** 10.1186/s13015-018-0125-4

**Published:** 2018-03-22

**Authors:** Samuele Girotto, Matteo Comin, Cinzia Pizzi

**Affiliations:** Department of Information Engineering, University of Padova, via Gradenigo 6/A, Padova, Italy

**Keywords:** Spaced seeds, K-mers, Efficient hashing

## Abstract

**Background:**

Patterns with wildcards in specified positions, namely *spaced seeds*, are increasingly used instead of *k*-mers in many bioinformatics applications that require indexing, querying and rapid similarity search, as they can provide better sensitivity. Many of these applications require to compute the hashing of each position in the input sequences with respect to the given spaced seed, or to multiple spaced seeds. While the hashing of *k*-mers can be rapidly computed by exploiting the large overlap between consecutive *k*-mers, spaced seeds hashing is usually computed from scratch for each position in the input sequence, thus resulting in slower processing.

**Results:**

The method proposed in this paper, fast spaced-seed hashing (FSH), exploits the similarity of the hash values of spaced seeds computed at adjacent positions in the input sequence. In our experiments we compute the hash for each positions of metagenomics reads from several datasets, with respect to different spaced seeds. We also propose a generalized version of the algorithm for the simultaneous computation of multiple spaced seeds hashing. In the experiments, our algorithm can compute the hashing values of spaced seeds with a speedup, with respect to the traditional approach, between 1.6$\times$ to 5.3$\times$, depending on the structure of the spaced seed.

**Conclusions:**

Spaced seed hashing is a routine task for several bioinformatics application. FSH allows to perform this task efficiently and raise the question of whether other hashing can be exploited to further improve the speed up. This has the potential of major impact in the field, making spaced seed applications not only accurate, but also faster and more efficient.

**Availability:**

The software FSH is freely available for academic use at: https://bitbucket.org/samu661/fsh/overview.

## Background

The most frequently used tools in bioinformatics are those searching for similarities, or local alignments, between biological sequences. *k*-mers, i.e. words of length *k*, are at the basis of many sequence comparison methods, among which the most widely used and notable example is BLAST [1].

BLAST uses the so-called “hit and extend” method, where a hit consists of a match of a 11-mers between two sequences. Then these matches are potential candidates to be extended and to form a local alignment. It can be easily noticed that not all local alignments include an identical stretch of length 11. As observed in [2] allowing for not consecutive matches increases the chances of finding alignments. The idea of optimizing the choice of the positions for the required matches, in order to design the so called *spaced seeds*, has been investigated in many studies, and it was used in PatternHunter [3], another popular similarity search software.

In general contiguous *k*-mers counts are a fundamental step in many bioinformatics applications [4–10]. However, spaced seeds are now routinely used, instead of contiguous *k*-mers, in many problems involving sequence comparison like: multiple sequence alignment [11], protein classification [12], read mapping [13] and for alignment-free phylogeny reconstruction [14]. More recently, it was shown that also metagenome reads clustering and classification can benefit from the use of spaced seeds [15–17].

A spaced seed of length *k* and weight $w<k$ is a string over the alphabet $\{1,0\}$ that contains *w* ‘1’ and $(k-w)$ ‘0’ symbols. A spaced seed is a mask where the symbols ‘1’ and ‘0’ denote respectively match and don’t care positions. The design of spaced seeds is a challenging problem itself, tackled by several studies in the literature [3, 18, 19]. Ideally, one would like to maximize the sensitivity of the spaced seeds, which is however an NP-hard problem [20].

The advantage of using spaced seeds, rather than contiguous *k*-mers, in biological sequence analysis, comes from the ability of such pattern model to account for mutations, allowing for some mismatches in predefined positions. Moreover, from the statistical point of view, the occurrences of spaced seeds at neighboring sequence positions are statistically less dependent than occurrences of contiguous *k*-mers [20]. Much work has been dedicated to spaced seeds over the years, we refer the reader to [21] for a survey on the earlier work.

Large-scale sequence analysis often relies on cataloging or counting consecutive *k*-mers in DNA sequences for indexing, querying and similarity searching. An efficient way of implementing such operations is through the use of hash based data structures, e.g. hash tables. In the case of contiguous *k*-mers this operation is fairly simple because the hashing value can be computed by extending the hash computed at the previous position, since they share $k-1$ symbols [22]. For this reason, indexing all contiguous *k*-mers in a string can be a very efficient process.

However, when using spaced seeds these observations do not longer hold. As a consequence, the use of spaced seeds within a string comparison method generally produces a slow down with respect to the analogous computation performed using contiguous *k*-mers. Therefore, improving the performance of spaced seed hashing algorithms would have a great impact on a wide range of bioinformatics tools.

For example, from a recent experimental comparison among several metagenomic read classifiers [23], Clark [7] emerged as one of the best performing tools for such a task. Clark is based on discriminative contiguous *k*-mers, and it is capable of classifying about 3.5M reads/min. When contiguous *k*-mers are replaced by spaced seeds, as in Clark-S [17], while the quality of the classification improves, the classification rate is reduced to just 200K reads/min.

The authors of Clark-S attributed such a difference to the use of spaced seeds. In particular, the possible sources of slowdown are two: the hashing of spaced seeds, and the use of multiple spaced seeds. In fact, Clark-S uses three different spaced seeds simultaneously in its processing. However, while the number of spaced seeds used could explain a 3$\times$ slowdown, running Clark-S is 17$\times$ slower than the original *k*-mer based Clark. Thus, the main cause of loss of speed performances can be ascribe to the use of spaced seed instead of contiguous *k*-mers. A similar reduction in time performance when using spaced seeds is reported also in other studies [12, 13, 15]. We believe that one of the causes of the slowdown is the fact that spaced seeds can not be efficiently hashed, as opposed to contiguous *k*-mers, raising the question of whether faster algorithms can be designed for this purpose.

In this paper we address the problem of the computation of spaced seed hashing for all the positions in an given input sequence, and present an algorithm that is faster than the standard approach to solve this problem. Moreover, since using multiple spaced seeds simultaneously on the same input string can increase the sensitivity [14], we also developed a variant of our algorithm for simultaneous hashing of multiple spaced seeds. Although faster implementations of specific methods that exploits spaced seeds are desirable, the main focus of this paper is the fast computation of spaced seed hashing.

In general, when computing a hash function there are also other properties of the resulting hash that might be of interest like: bit dependencies, hash distributions, collisions etc. However, the main focus of this paper is the fast computation of spaced seed hashing, using the simple Rabin-Karp rolling hash function. It is important to observe that many hashing functions can be efficiently computed from the Rabin-Karp rolling hash. For example, our method can be extended to implement the cyclic polynomial hash used in [22] with no extra costs.

In the "Methods" section we briefly summarize the properties of spaced seeds and describe our algorithm, FSH,^1^ together with a variant for handling multiple seed hashing. Then, experimental results on NGS reads hashing for various spaced seeds are reported and discussed.

## Methods

A *spaced-seed S* (or just a seed) is a string over the alphabet $\{1,0\}$ where the 1s correspond to matching positions. The *weight* of a seed corresponds to the number of 1s, while the overall *length*, or span, is the sum of the number of 0s and 1s.

Another way to denote a spaced seed is through the notation introduced in [25]. A spaced seed can be represented by its *shape Q* that is the set of non negative integers corresponding to the positions of the 1s in the seed. A seed can be described by its shape *Q* where its weight *W* is denoted as |*Q*|, and its span *s*(*Q*) is equal to $\max Q + 1$. For any integer *i* and shape *Q*, the positioned shape $i+Q$ is defined as the set $\{i+k, k \in Q\}$. Let us consider the positioned shape $i+Q=\{i_0,i_1,\dots ,i_{W-1}\}$, where $i=i_0<i_1<...<i_{W-1}$, and let $x=x_0 x_1 \dots x_{n-1}$ be a string over the alphabet $\mathcal {A}$. For any position *i* in the string *x*, with $0\le i \le n-s(Q)$, the positioned spaced seed $i+Q$ identifies a string of length |*Q*| that we call *Q*-gram. A *Q*-gram at position *i* in *x* is the string $x_{i_0} x_{i_1} \dots x_{i_{W-1}}$ and it is denoted by $x[i+Q]$.

### Example

Let $Q=\{0,2,3,4,6,7\}$, then *Q* is the seed 10111011, its weight is $|Q|=6$ and its span is $s(Q)=8$. Let us consider the string $x=ACTGACTGGA$, then the *Q*-gram $x[0+Q]=ATGATG$ can be defined as:

$$\begin{aligned} \begin{array}{lllllllllll} {\text {x}} &{} ~~{\text {A}} &{} ~~{\text {C}} &{} ~~{\text {T}} &{} ~~{\text {G}} &{} ~~{\text {A}} &{} ~~{\text {C}} &{} ~~{\text {T}} &{} ~~{\text {G}} &{} ~~{\text {G}} &{} ~~{\text {A}} \\ {\text {Q}} &{} ~~{\text {1}} &{} ~~{\text {0}} &{} ~~{\text {1}} &{} ~~{\text {1}} &{} ~~{\text {1}} &{} ~~{\text {0}} &{} ~~{\text {1}} &{} ~~{\text {1}} &{} ~~{} &{} ~~{} \\ {{\text {x[0 + Q]}}} &{} ~~{\text {A}} &{} {} &{} ~~{\text {T}} &{} ~~{\text {G}} &{} ~~{\text {A}} &{} ~~{} &{} ~~{\text {T}} &{} ~~{\text {G}} &{} ~~{} &{} ~~{} \end{array} \end{aligned}$$

Similarly all other *Q*-grams are $x[1+Q]=CGACGG$, and $x[2+Q]=TACTGA$.

### Spaced seed hashing

In order to hash any string, first we need to have a coding function from the alphabet $\mathcal {A}$ to a binary codeword. For example let us consider the function $encode: \mathcal {A} \rightarrow \{0,1\}^{log_2|\mathcal {A}|}$, with the following values $encode(A)=00, encode(C)=01, encode(G)=10, encode(T)=11$. Based on this function we can compute the encodings of all symbols of the *Q*-gram $x[0+Q]$ as follows:

$$\begin{aligned} \begin{array}{lllllll} {x{\text {[0 + Q]}}} &{} ~~{\text {A}} &{} ~~{\text {T}} &{} ~~{\text {G}} &{} ~~{\text {A}} &{} ~~{\text {T}} &{} ~~{\text {G}}\\ {encodings} &{} ~~{{\text {00}}} &{} ~~{{\text {11}}} &{} ~~{{\text {10}}} &{} ~~{{\text {00}}} &{} ~~{{\text {11}}} &{} ~~{{\text {10}}}\\ \end{array} \end{aligned}$$

There exist several hashing functions, in this paper we consider the Rabin-Karp rolling hash, defined as $h(x[0+Q])=encode(A)*|\mathcal {A}|^0+encode(T)*|\mathcal {A}|^1+encode(G)*|\mathcal {A}|^2+encode(A)*|\mathcal {A}|^3+encode(T)*|\mathcal {A}|^4+encode(G)*|\mathcal {A}|^5$. In the original Rabin-Karp rolling hash all math is done in modulo *n*, here for simplicity we avoid that. In the case of DNA sequences $|\mathcal {A}|=4$, that is a power of 2 and thus the multiplications can be implemented with a shift. In the above example, the hashing value associated to the *Q*-gram *ATGATG* simply corresponds to the list of encoding in Little-endian: 101100101100.

To compute the hashing value of a *Q*-gram from its encodings one can define the function $h(x[i+Q])$, for any given position *i* of the string *x*, as:

1 $$\begin{aligned} h(x[i+Q]) = \bigvee _{k \in Q} ( encode(x_{i+k}) \ll m(k)*log_2|\mathcal {A}| ) \end{aligned}$$

Where *m*(*k*) is the number of shifts to be applied to the encoding of the *k*-th symbols. For a spaced seed *Q* the function *m* is defined as $m(k)=|\{i\in Q, \text{ such } \text{ that } i < k\}|$. In other words, given a position *k* in the seed, *m* stores the number of matching positions that appear to the left of *k*. The vector *m* is important for the computation of the hashing value of a *Q*-gram.

#### Example

In the following we report an example of hashing value computation for the *Q*-gram $x[0+Q]$.

**Table:** 

x	A	C	T	G	A	C	T	G	G	A
Q	1	0	1	1	1	0	1	1		
m	0	1	1	2	3	4	4	5		
Shifted-encodings	00		11 $\ll$ 2	10 $\ll$ 4	00 $\ll$ 6		11 $\ll$ 8	10 $\ll$ 10		
			1100							
				101100						
					00101100					
							1100101100			
Hashing value								101100101100		

The hashing values for the others *Q*-grams can be determined through the function $h(x[i+Q])$ with a similar procedure. Following the above example the hashing values for the *Q*-grams $x[1+Q]=CGACGG$ and $x[2+Q]=TACTGA$ are respectively 101001001001 and 001011010011.

In this paper we decided to use the Rabin-Karp rolling hash, because it is very intuitive. There are other hashing functions, like the cyclic polynomial hash, that are usually more appropriate because of some desirable properties like uniform distribution in the output space, universality, higher-order independence [22]. In this paper we will focus on the efficient computation of the Rabin-Karp rolling hash. However, with the same paradigm proposed in the following sections, one can compute also the cyclic polynomial hash by replacing: shifts with rotations, OR with XOR, and the function *encode*(*A*) in Eq. (1) with a seed table where the letters of the DNA alphabet are assigned different random 64-bit integers.

### Fast spaced seed hashing

In many applications [11–15, 17] it is important to scan a given string *x* and to compute the hashing values over all positions. In this paper we want to address the following problem.

#### **Problem 1**

Let us consider a string $x= x_0 x_1 \ldots x_i \ldots x_{n-1}$, of length *n*, a spaced seed *Q* and an hash function *h* that maps strings into a binary codeword. We want to compute the hashing values $\mathcal {H}(x,Q)$ for all the *Q*-grams of *x*, in the natural order starting from the first position 0 of *x* to the last $n-s(Q)$.

$$\begin{aligned} \mathcal {H}(x,Q) = \langle h(x[0+Q]), h(x[1+Q]), \dots h(x[n-s(Q)]) \rangle \end{aligned}$$

Clearly, in order to address Problem 1, it is possible to use Eq. 1 for each position of *x*. Note that, in order to compute the hashing function $h(x[i+Q])$ for a given position, the number of symbols that have to be extracted from *x* and encoded into the hash is equal to the weight of the seed |*Q*|. Thus such an approach can be very time consuming, requiring the encoding of $|Q|(n-s(Q))$ symbols. In summary, loosely speaking, in the above process each symbol of *x* is read and encoded into the hash |*Q*| times.

In this paper we present a solution for Problem 1 that is optimal in the number of encoded symbols. The scope of this study is to minimize the number of times that a symbol needs to be read and encoded for the computation of $\mathcal {H}(x,Q)$. Since the hashing values are computed in order, starting from the first position, the idea is to speed up the computation of the hash at a position *i* by reusing part of the hashes already computed at previous positions.

As mentioned above, using Eq. 1 in each position of an input string *x* is a simple possible way to compute the hashing values $\mathcal {H}(x,Q)$. However, we can study how the hashing values are built in order to develop a better method. For example, let us consider the simple case of a contiguous *k*-mers. Given the hashing value at position *i* it is possible to compute the hashing for position $i+1$, with three operations: a rotation, the deletion of the encoding of the symbol at position *i*, and the insertion of the encoding of the symbol at position $i+k$, since the two hashes share $k-1$ symbols. In fact in [22] the authors showed that this simple observation can speed up the hashing of a string by recursively applying these operations. However, if we consider the case of a spaced seed *Q*, we can clearly see that this observation does not hold. In fact, in the above example, two consecutive *Q*-grams, like $x[0+Q]=ATGATG$ and $x[1+Q]=CGACGG$, do not necessarily have much in common.

In the case of spaced seeds the idea of reusing part of the previous hash to compute the next one needs to be further developed. More precisely, because of the shape of a spaced seed, we need to explore not only the hash at the previous position, but all the $s(Q)-1$ previous hashes.

Let us assume that we want to compute the hashing value at position *i* and that we already know the hashing value at position $i-j$, with $j<s(Q)$. We can introduce the following definition of $\mathcal {C}_j = \{k-j \in Q: k \in Q \wedge m(k-j) = m(k)-m(j)\}$ as the positions in *Q* that after *j* shifts are still in *Q* with the propriety of $m(k-j) = m(k)-m(j)$. In other words, if we are processing the position *i* of *x* and we want to reuse the hashing value already computed at position $i-j$, $\mathcal {C}_j$ represents the symbols of $h(x[i-j+Q])$ that we can keep while computing $h(x[i+Q])$. More precisely, we can keep the encoding of $|\mathcal {C}_j|$ symbols from that hash and insert the remaining $|Q| - |\mathcal {C}_j|$ symbols at positions *Q* $\backslash$ $C_j$.

#### Example

If we know the first hashing value $h(x[0+Q])$ and we want to compute the second hash $h(x[1+Q])$, the following example show how to construct $C_1$.

**Table:** 

k		0	1	2	3	4	5	6	7
Q		1	0	1	1	1	0	1	1
Q$\ll$1	1	0	1	1	1	0	1	1	
m(k)		0	1	1	2	3	4	4	5
m(k) − m(1)	− 1	0	0	1	2	3	3	4	
$C_1$				2	3			6	

The symbols at positions $C_1=\{2,3,6\}$ of the hash $h(x[1+Q])$ have already been encoded in the hash $h(x[0+Q])$ and we can keep them. In order to complete $h(x[1+Q])$, the remaining $|Q| - |\mathcal {C}_1|= 3$ symbols need to be read from *x* at positions $i+k$, where $i=1$ and $k \in Q \backslash C_1 = \{0,4,7\}$.

**Table:** 

x	A	C	T	G	A	C	T	G	G	A
$x[0+Q]$	A		T	G	A		T	G		
$C_1$				2	3			6		
$Q \backslash C_1$		0				4			7	
$x[1+Q]$		C		G	A	C		G	G	

Note that the definition of $|\mathcal {C}_j|$ is not equivalent to the overlap complexity of two spaced seeds, as defined in [19]. In some cases, like the one presented above, the overlap complexity coincides with $|\mathcal {C}_1|=3$. However, there are other cases where $|\mathcal {C}_j|$ is smaller than the overlap complexity.

#### Example

Let us consider the hash at position 2 $h(x[2+Q])$, and the hash at position 0 $h(x[0+Q])$. In this case we are interested in $\mathcal {C}_2$.

**Table:** 

k			0	1	2	3	4	5	6	7
Q			1	0	1	1	1	0	1	1
Q $\ll$ 2	1	0	1	1	1	0	1	1		
m(k)			0	1	1	2	3	4	4	5
m(k) − m(2)	− 1	0	0	1	2	3	3	4		
$C_2$			0				4			

The only symbols that can be preserved from $h(x[0+Q])$ in order to compute $h(x[2+Q])$ are those at positions 0 and 4, whereas the overlap complexity is 3.

For completeness we report all values of $\mathcal {C}_j$:

$$\begin{aligned} \mathcal {C}&=\langle \mathcal {C}_1, \ldots , \mathcal {C}_7\rangle \\ &= \langle\{2,3,6\}, \{0,4\}, \{0,3,4\}, \{0,2,3\}, \{2\}, \{0\}, \{0\}\rangle \end{aligned}$$

In order to address Problem 1, we need to find, for a given position *i*, the best previous hash that ensures to minimize the number of times that a symbol needs to be read and encoded, in order to compute $h(x[i+Q])$. We recall that $|\mathcal {C}_j|$ represents the number of symbols that we can keep from the previous hash at position $i-j$, and thus the number of symbols that needs to be read and encoded are |*Q* $\backslash$ $C_j|$. To solve Problem 1 and to minimize the number of symbols that needs to be read, |*Q* $\backslash$ $C_j|$, it is enough to search for the *j* that maximizes $|\mathcal {C}_j|$. The best previous hash can be detected with the following function:

$$\begin{aligned} ArgBH(s)= \arg \max _{j\in [1,s]} |\mathcal {C}_j| \end{aligned}$$

If we have already computed the previous *j* hashes, the best hashing value can be found at position $i-ArgBH(j)$, and will produce the maximum saving $|\mathcal {C}_{ArgBH(j)}|$ in terms of symbols that can be kept. Following the above observation we can compute all hashing values $\mathcal {H}(x, Q)$ incrementally, by using dynamic programming as described by the pseudocode of FSH.
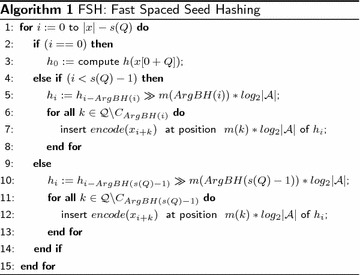


The above dynamic programming algorithm, FSH, scans the input string *x* and computes all hashing value according to the spaced seed *Q*. In order to better understand the amount of savings we evaluate the above algorithm by counting the number of symbols that are read and encoded. First, we can consider the input string to be long enough so that we can discard the transient of the first $s(Q)-1$ hashes. Let us continue to analyze the spaced seed 10111011. If we use the standard function $h(x[i+Q])$ to compute all hashes, each symbol of *x* is read $|Q|=6$ times. With our algorithm, we have that $|\mathcal {C}_{ArgBH(7)}|=3$ and thus half of the symbols do need to be encoded again, overall each symbol is read three times. The amount of saving depends on the structure of the spaced seed. For example, the spaced seed 10101010101, with the same weight $|Q|=6$, is the one that ensures the best savings ($|\mathcal {C}_{ArgBH(10)}|=5$). In fact, with our algorithm, we can compute all hashing values while reading each symbol of the input string only once, as with contiguous *k*-mers. To summarize, if one needs to scan a string with a spaced seed and to compute all hashing values, the above algorithm guarantees to minimize the number of symbols to read.

### Fast multiple spaced seed hashing

Using multiple spaced seeds, instead of just one spaced seed, is reported to increase the sensitivity [14]. Therefore, applications that exploit such an observation (for example [15–17, 26]) will benefit from further speedup that can be obtained from the information already computed from multiple spaced seeds.

Our algorithm, FSH, can be extended to accommodate the need of hashing multiple spaced seeds simultaneously, without backtracking. Let us assume that we have a set $S={s_1,s_2,..., s_{|S|}}$ of spaced seeds, all of the same length *L*, from which we can compute the corresponding vectors $m_{s_i}$. To this purpose, FSH needs to be modified as follows. First of all, a new cycle (between steps 2 and 14) is needed to iterate the processing among the set of all spaced seeds. Next, $\mathcal {C}_j$ needs to be redefined so that it compares not only a given spaced seed with itself, but all spaced seeds vs all:

$$\begin{aligned} \mathcal {C}^{yz}_j = \{k-j \in s_y: k \in s_z \wedge m_{s_y}(k-j) = m_{s_z}(k)-m_{s_z}(j)\} \end{aligned}$$

In the new definition $\mathcal {C}^{yz}_j$ evaluates the number of symbols in common between the seed $s_y$ and the *j*-th shift of the seed $s_z$. The function $\mathcal {C}^{yz}_j$ allows to identify, while computing the hash of $s_y$, the number of symbols in common with the *j*-th shift of seed $s_z$. Similarly, we need to redefine *ArgBH*(*i*) so that it detects not only the best previous hash, but also the best seed. We define

$$\begin{aligned} ArgBSH(y,s)= \arg \max _{z \in [1,|S|], j\in [1,s] } |\mathcal {C}^{yz}_j| \end{aligned}$$

that returns, for the seed $s_y$, the pair $(s_z,p)$ representing the best seed $s_z$ and best hash *p*. With these new definitions we can now adjust our algorithm so that, while computing the hash of $s_y$ for a given position *i*, it can start from the best previous hash identified by the pair $ArgBSH(y,s)=(s_z,p)$. The other steps for the insertion of the remaining symbols do not need to be modified.

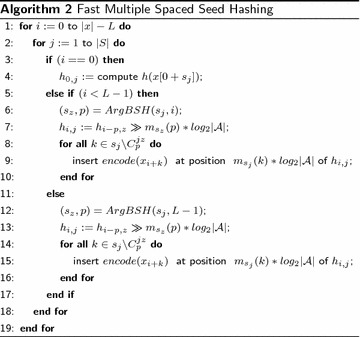



## Results and discussion

In this section we will discuss the improvement in terms of time speedup of our approach ($T_{FSH}$) with respect to the time $T_{Eq1}$ needed for computing spaced seeds hashing repeatedly using Eq. 1: $\text{ speedup } = \frac{T_{Eq1}}{T_{FSH}}$.

### Spaced seeds and datasets description

The spaced seeds we used have been proposed in literature as maximizing the hit probability [17], minimizing the overlap complexity [18] and maximizing the sensitivity [18]. We tested nine of such spaced seeds, three for each category. The spaced seeds are reported in Table 1 and labeled Q1, Q2, ...,Q9. Besides these spaced seeds, we also tested Q0, which corresponds to an exact match with a 22mer (all 22 positions are set to 1), and Q10, a spaced seed with repeated ‘10’ and a total of 22 symbols equal to ‘1’. All spaced seeds $Q0-Q10$ have the same weight $|Qi|=22$. Furthermore, in order to compare seeds with different density, we computed with rasbhari several sets of seeds with weights from 11 to 32 and lengths from 16 to 45.

**Table 1:** The nine spaced seeds used in the experiments grouped according to their type

Spaced seeds maximizing the hit probability [17]	
Q1	1111011101110010111001011011111
Q2	1111101011100101101110011011111
Q3	1111101001110101101100111011111
Spaced seeds minimizing the overlap complexity [18]	
Q4	1111010111010011001110111110111
Q5	1110111011101111010010110011111
Q6	1111101001011100111110101101111
Spaced seeds maximizing the sensitivity [18]	
Q7	1111011110011010111110101011011
Q8	1110101011101100110100111111111
Q9	1111110101101011100111011001111

The datasets we used were taken from previous scientific papers on metagenomic read binning and classification [6, 27]. We considered both simulated datasets (S,L,R), and synthetic datasets (MiSeq, HiSeq, MK_a1, MK_a2, and simBA5). The datasets $S_x$ and $L_x$ contain sets of paired-end reads of length approximately 80 bp generated according to the Illumina error profile with an error rate of 1%, while the datasets $R_x$ contain Roche 454 single-end long reads of length approximately 700bp, and a sequencing error of 1%. The synthetic datasets represent mock communities built from real shotgun reads of various species. Table 2 shows, for each dataset, the number of reads and their average length.

**Table 2:** Number of reads and average lengths for each of the dataset used in our experiments

Datasets	Number of reads	Avg. read length
S6	1,426,457	80
S7	3,307,100	80
S9	4,468,336	80
S10	9,981,172	80
L5	1,016,418	80
L6	1,182,178	80
HiSeq	9,989,713	91
simBA5	5,439,738	100
MixK1	9,629,886	101
MixK2	7,149,900	101
MiSeq	9,933,556	131
R7	290,473	702
R8	374,576	715
R9	588,256	715

All the experiments where run on a laptop equipped with an Intel i74510U cpu at 2 GHz, and 16 GB RAM.

### Analysis of the time performances

**Fig. 1:**
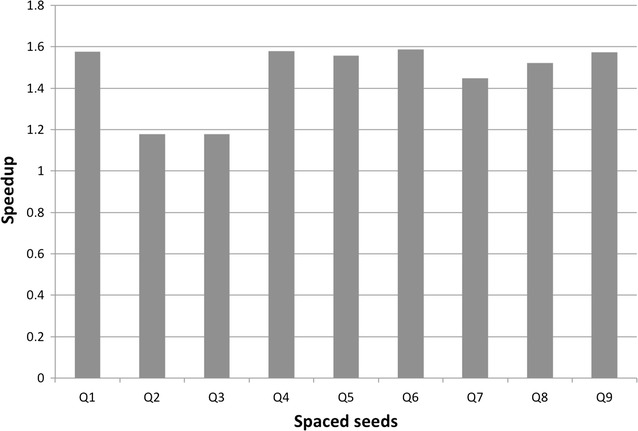
The speedup of our approach with respect to the standard hashing computation, as a function of the spaced seeds used in our experiments

Figure 1 plots, for each spaced seed, the speedup that is obtainable with our approach with respect to the standard hashing computation. As a reference, the baseline given by the standard approach is about 17 min to compute the hash for a given seed on all datasets.

First of all it can be noticed that our approach improves over the standard algorithm for all of the considered spaced seeds. The smallest improvements are for the spaced seeds Q2 and Q3, both belonging to the class of spaced seeds maximizing the hit probability, for which the speedup is almost 1.2$\times$, and the running time is about 15 min. For all the other spaced seeds the speedup is close to 1.6$\times$, thus saving about 40% of the time required by the standard computation, and ending the computation in less than 11 min on average.

**Fig. 2:**
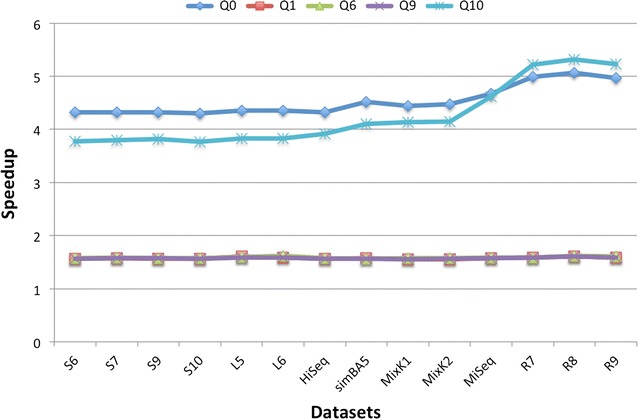
Details of the speedup on each of the considered datasets. Q0 is the solid 22mer, Q10 is the spaced seed with repeated 10. The other reported spaced seeds are the ones with the best performances for each class: Q1 (maximizing the hit probability), Q6 (minimizing the overlap complexity) and Q9 (maximizing the sensitivity)

Figure 2 shows the performances of our approach with respect to the single datasets. In this experiment we considered the best performing spaced seed in each of the classes that we considered, namely Q1, Q6, and Q9, and the two additional special cases Q0 and Q10.

We notice that for the spaced seeds Q0 and Q10 the standard approach requires respectively, 12 and 10 min, to process all datasets. This is already an improvement of the standard method with respect to the 17 min required with the other seeds $Q1-Q9$. Nevertheless, with our algorithm the hashing of all dataset can be completed in just 2.7 min for Q0 e 2.5 min for Q10, with a speedup of 4.5$\times$ and 4.2$\times$.

We observe that while the speedup for the spaced seeds Q1, Q6, and Q9 is basically independent on the dataset and about 1.6$\times$, the speedup for both the 22-mer Q0 and the ‘alternate’ spaced seed Q10 is higher, spanning from 4.3$\times$ to 5.3$\times$, depending on the seed and on the dataset. In particular, the speedup increases with the length of the reads and it achieves the highest values for the long read datasets $R_7, R_8$ and $R_9$. This behavior is expected, as these datasets have longer reads with respect to the others, thus the effect of the initial transient is mitigated.

### Multiple spaced seed hashing

When the analysis of biological data to perform requires the use of multiple spaced seeds, it is possible to compute the hash of all seeds simultaneously while reading the input string with the method described in Section.

**Fig. 3:**
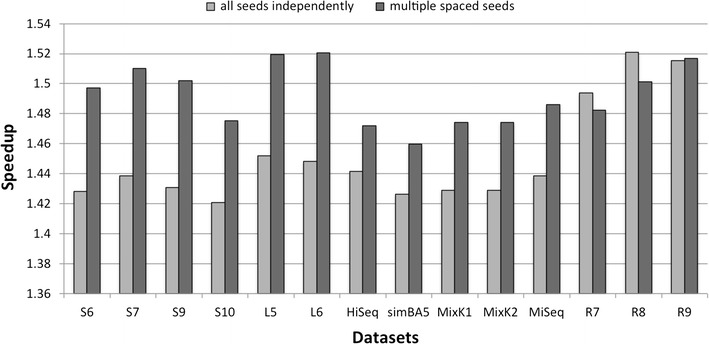
Details of the time speedup of our approach with the multiple spaced seeds hashing (dark grey) and of our approach with each spaced seed hashed independently (light grey)

In Fig. 3 we report the comparison between the speedup we obtained when computing the hash for each spaced seed Q1,...,Q9 independently (light grey), and the speedup we obtained when using the multiple spaced seeds approach (dark grey).

In most cases, multiple spaced seed hashing allows for a further improvement of about 2–5%, depending on the dataset. In terms of absolute values, the standard computation to hash all datasets requires 159 min, the computation of all seeds independently with the approach described in Section takes 109 min, while the simultaneous computation of multiple spaced seeds with our method takes 107 min. When considering all datasets the average speedup increases from 1.45$\times$ (independent computation) to 1.49$\times$ (simultaneous computation). The small improvement can be justified by the fact that the spaced seeds considered are by construction with minimal overlap.

### Predicted speedup vs real speedup

**Fig. 4:**
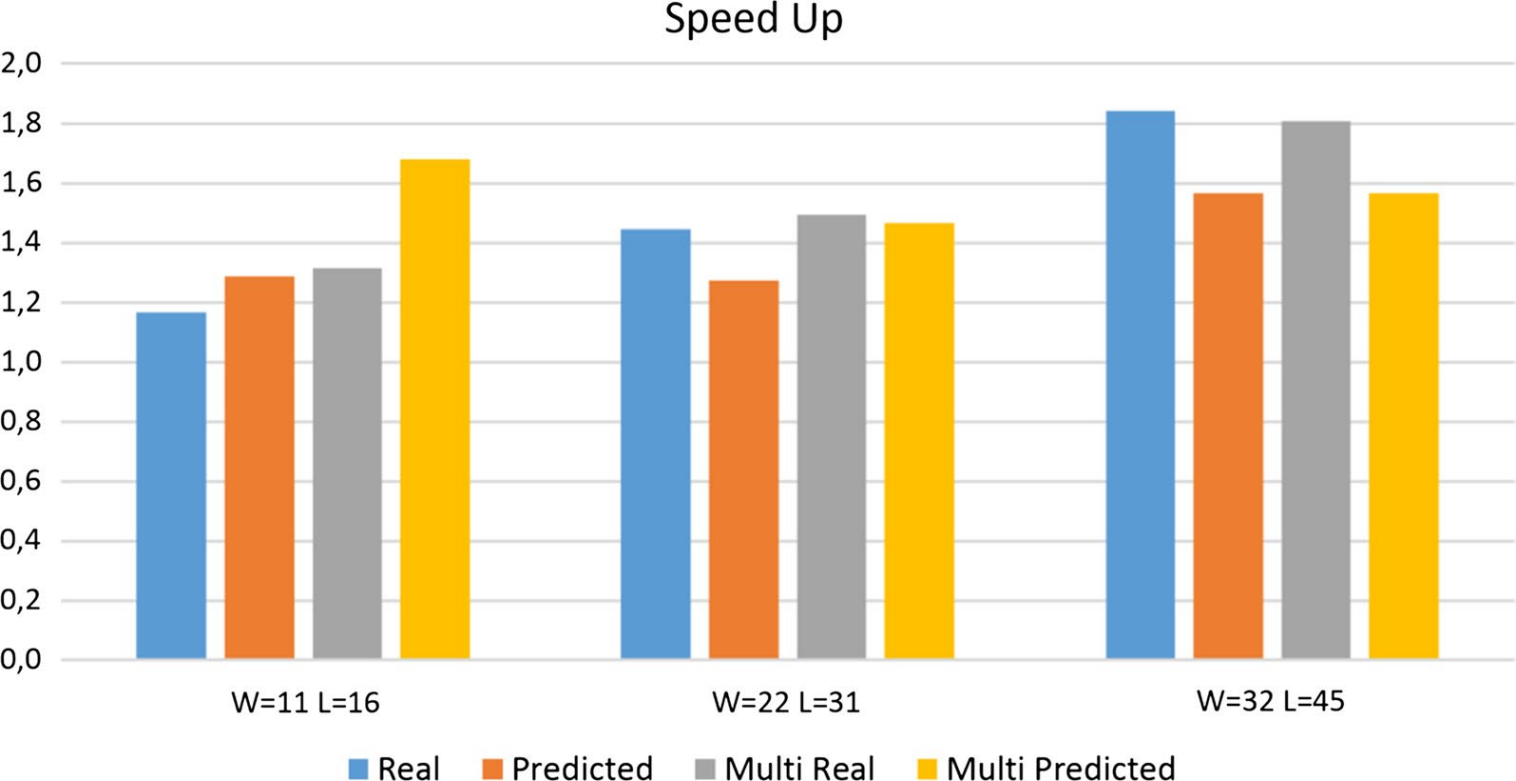
The theoretical and real speedup of our approach with respect to the standard hashing computation, as a function of the spaced seeds weight

In Fig. 4 are reported the average speedup (Real), over all datasets, for the three different groups of nine seeds with the same density (W/L), generated with rasbhari [18]. In the same Figure we include also the speedup when all nine seeds are used simultaneously (Multi) and the theoretical speedup predicted by our method (Predicted).

As, for the theoretical predicted speedups, these are usually in line with the real speedups even if the absolute values are not necessarily close. We suspect that the model we use, where shifts and insertions have the same cost, is too simplistic. Probably, the real computational cost for the insertion of a symbol is greater than the cost for shifting, and also cache misses might play a role.

If the theoretical speedup for multiple seeds is greater than the theoretical speedup for independent seeds, this indicates that in principle, with multiple seeds, it is possible to improve with respect to the computation of seeds independently. It is interesting to note that the real results confirm these predictions. For example, in the multiple seeds with weights 32, it is impossible to improve both theoretically and in practice. In the other two cases, the computation of multiple seeds is faster in practice as correctly predicted by the theoretical speedup.

### The effect of spaced seeds weight and reads length

**Fig. 5:**
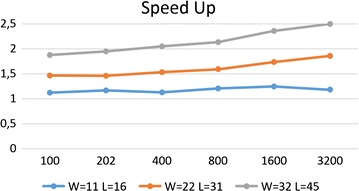
The speedup of our approach with respect to the standard hashing computation as a function of reads length and the spaced seeds weight (all with the same density)

To better understand the impact of reads length and density of spaced seeds on the speedup, in this section we report a series of experiments under various conditions. In order to compare the performance of our method on spaced seeds with different weights we generated several sets of nine spaced seeds with rasbhari [18] with weights from 11 to 32 and lengths from 16 to 45. First, we test how the reads length affects the speedup. In Fig. 5 we report the speedup as a function of the reads length, for various spaced seeds with the same density (*W* / *L*).

We can observe that the speedup increases as a function of the reads length. This is expected, in fact the effect of the initial transient of our hashing computation is mitigated on longer reads. Another interesting behavior is the fact that, although the spaced seeds have all the same density, longer spaced seeds have the highest speedup. A possible explanation lies in the way our algorithm works. Since our hashing computation explores the previous *L* hashes searching for redundancies, as the length of the spaced seed increases, also our ability to reuse the previous hashes increases, and similarly it does the speedup.

**Fig. 6:**
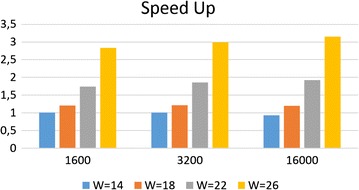
The speedup of our approach with respect to the standard hashing computation as a function of reads length and the spaced seeds density (L=31 and W varies)

In Fig. 6 we compare the speedup of various spaced seeds as a function of the weight W, while the length $L=31$ remains constant.

We can note that if the weight of the seeds grows then also the speedup grows. This behavior is observed for various reads length. This phenomenon can be explained as follows, if a spaced seed has more 1s (higher weight), then the chances to reuse part of the seed increase, and consequently the speedup of FSH increases.

## Conclusions and future work

In this paper we tackle the problem of designing faster algorithms for the computation of spaced seed hashing. We presented a new approach, FSH, for spaced seeds hashing that exploits the information from adjacent hashes, in order to minimize the operations that need to be performed to compute the next hash. In summary, FSH can speedup spaced seed hashing on various conditions. The experiments we performed, on short NGS reads, showed that FSH has a speedup of 1.6$\times$, with respect to the standard approach, for several kind of spaced seeds defined in the literature. Furthermore, the gain greatly improved in special cases, where seeds show a high autocorrelation, and for which a speed up of about 4$\times$ to 5$\times$ can be achieved. The benefit in terms of computation time increases as the length of the reads grows, like in modern sequencing technologies, or when long and complex spaced seeds are needed.

Another contribution of this work is to open the way to the development of further research on methods for speeding up spaced seed hashing computation. In the future, we plan to investigate alternative ways to compute spaced seed hashing based on indexing strategies. Another interesting direction of research is to experimentally evaluate the impact of fast spaced seed hashing in different bioinformatics contexts where tools based on spaced seeds are used.

## Footnotes

1 a preliminary version of this manuscript was published in [24].
